# Exploring the effect of Covid-19 on herding in Asian financial markets

**DOI:** 10.1016/j.mex.2022.101961

**Published:** 2022-12-05

**Authors:** C.T. Vidya, Rashika Ravichandran, Aditya Deorukhkar

**Affiliations:** a Centre for Economic and Social studies (CESS), Begumpet, Hyderabad, India; b Christ University, Bengaluru, India

**Keywords:** Covid-19, Herding, Investor behaviour, Asian economies, Market returns, Anti-herding, Cross-Sectional Absolute Deviation (CSAD)

## Abstract

We examine herding behavior before, during, and after the Covid-19 pandemic in eight prominent Asian stock markets. Daily stock returns for the period Jan- 2018 to July- 2022 in the markets were investigated using the models prescribed by Chang et al., (2000) and Chiang and Zheng (2010). The empirical results provide strong support to earlier studies by providing robust evidence of herding in Vietnam, Indonesia, India, South Korea, and Singapore when the market is bullish and Indonesia and Vietnam also exhibit herding when the market is bearish. Herding tendency is dominant for Vietnam, India, and Indonesia during the pandemic with the post-pandemic time being more potent for China and Vietnam. Notably, an anti-herding tendency is found in China, Hong Kong, and Singapore. As a policy measure, efficient information dissemination, deterrence of insider trading, and regulation of mispricing can be undertaken.

## Highlights

• We conduct an in-depth analysis of the Asian financial markets in bullish and bearish markets in pre, during, and post-COVID periods.
• Using Cross-Sectional Absolute Deviation (CSAD) in market return, we find varying herding and anti-herding patterns during the crisis.
• We also show the implications and the effects of herd formation on the markets.

## Graphical abstract

**Figure:**
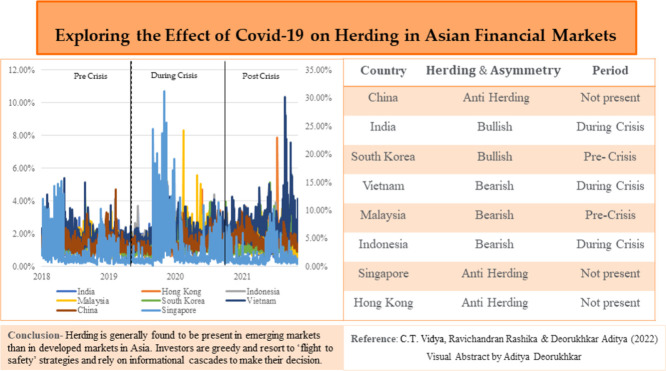

Specification Table

**Table:** 

Subject area	Economics and Finance
More specific subject area	*Behavioral Finance*
Name of your method	*Cross-Sectional Absolute Deviation (CSAD)*
Name and reference of original method	• *Chang, E. C., Cheng, J. W., & Khorana, A. (2000). An examination of herd behavior in equity. Journal of Banking & Finance, 24, 1651 - 1679.* • *Chiang, T., & Zheng, D. (2010). An empirical analysis of herd behavior in global stock markets. Journal of Banking & Finance, 34(8), 1911-1921.*
Resource availability	• *Data is included in this publication* • *Results can be reproduced using any economic/statistical software like EViews, Stata, R, Python, etc.*

## Introduction

In February 2020, the outbreak of COVID-19 caused an unprecedented crisis in economies and these gyrations were being red in the stocks’ prices. The investors seemed to be on a roller-coaster ride with their expectations and anxiety taking the form of irrational decisions due to abnormality being present [[1], [2]–3]. Financial market volatility drives investors to follow the crowd by manifesting into herding [4]. Herding is a situation in which investors ‘decisions influence the collective behaviour of the market and they act irrationally thereby turning the market inefficient. Documenting herding behaviour has reported mixed trends for the developed and developing markets [[5], [6]–7]. The Asian financial markets are one of the largest in the world and Asians are known to suffer from cognitive and behavioral biases on a different level as compared to other cultures such as European and Latin American [8]. Thus, studying herding in Asia can give interesting results concerning herd propensity and the impact of exogenous informational cascades on investors' rationality. We also study herding during tranquil and turbulent times and conclusively prove that investors in developing markets can be motivated by greed due to their propensity to form herds during bullish market conditions.

In this paper, we study herding amongst individual investors in Asian countries - India, China, Vietnam, Indonesia, Malaysia, Singapore, Hong Kong, and South Korea. We use the model of Chiang and Zheng (2010) to investigate the effects of herding in *normal marke*t conditions and a model used in Chang et al., (2000) to study *extreme market* conditions while providing evidence regarding the prevalence of the “anti-herding” sentiment.

Our findings contribute to a growing literature on the financial market effect of COVID-19. The following studies examined how COVID-19 has influenced the financial market [[9], [10]–11] on global trade [[12], [13]–14] on the aggregate market sentiments and impact on stock markets specifically [[15], [16], [17], [18], [19]–20]

Our study also contributes to the expanding body of research examining the pandemic's impact on investor behavior and challenging decisions while connecting it with market movements across various Asian markets. In addition, it reports findings of any asymmetries in the market movements across markets and proves if stock exchanges are resilient to global financial shocks.

## Methods

In this section, we discuss the methodology we have adopted to investigate the presence of herding. Our sample consists of daily stock market data from Jan’2018 to July’2022. We use the following model Chiang by and Zheng (2010) to comprehend the presence of herd behavior in the markets.

(1) $$CSAD_{t\;}\;=\;\alpha \;+\gamma_{1}R_{m,t}+\;\gamma_{2}\left|R_{m,t}\right|\;+\;\gamma_{3}{\left(R_{m,t}\right)}^{2}\;+\;\varepsilon_{t}$$

Where γ_1_ is the linear co-efficient between market return and CSAD, γ_2_ is the non-linear coefficient between absolute market return and CSAD. $\gamma_{3}\;$relates cross-sectional absolute deviation to squared market return and checks for nonlinear dynamics of herding and if herding is present then this coefficient would be negative and statistically significant.

To measure herding that occurs in extreme market conditions and its direction we use the following models outlined in Chang et al., (2000)

(2) $$CSAD_{t}^{UP}=\;\alpha +\gamma_{1}^{UP}\left|R_{m,t}^{UP}\right|+\gamma_{2}^{UP}{\left(R_{m,t}^{UP}\right)}^{2}+\varepsilon_{t}$$

(3) $$CSAD_{t}^{DOWN}=\;\alpha +\gamma_{1}^{DOWN}\left|R_{m,t}^{DOWN}\right|+\gamma_{2}^{DOWN}{\left(R_{m,t}^{DOWN}\right)}^{2}+\varepsilon_{t}$$

Here, $CSAD_{t}$ is the average absolute deviation of each stock compared to the return of the market ($R_{m,t}$) in period t. The negative and statistically significant estimated coefficients $\gamma_{2}^{UP}$and $\gamma_{2}^{DOWN}\;$respectively, indicate presence of herding during bullish and bearish markets and if the magnitudes are significant then we can conclude that herding behaviour is asymmetric in both up and down markets.

To test for herding behavior during tranquil and turbulent times, we divide our dataset into pre-covid, during-covid, and post-covid subsets to examine the presence of herding using Eq. (1) in all the countries and to test for anti-herding sentiment we adopt the methodology proposed by Babalos and Stavroyiannis (2015) where they opined that if anti-herding is present then $\gamma_{3}>0$ in Eq. (1).

## Results

**Fig. 1:**
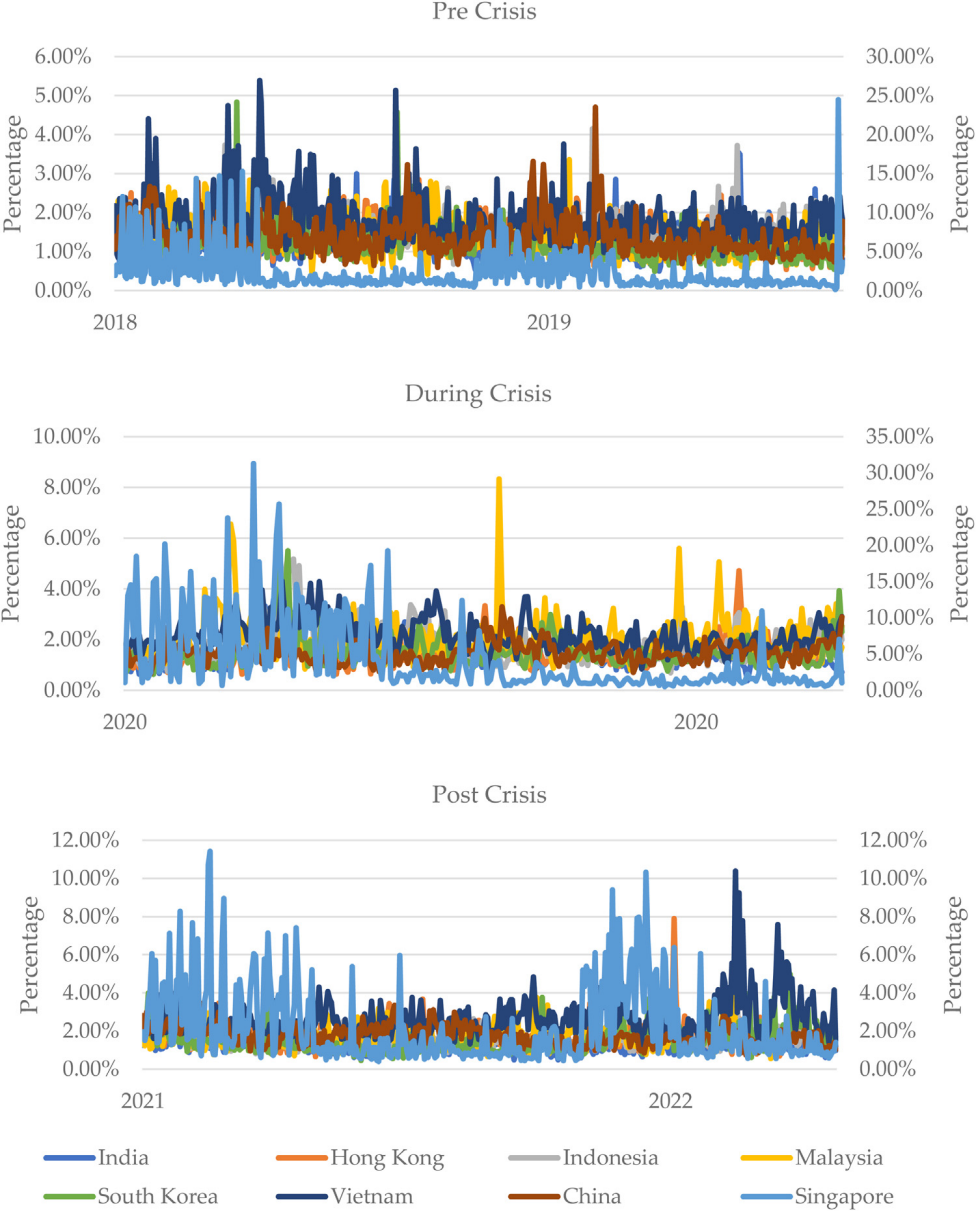
Trends in Herding during Pre, During and Post Covid-19 Crisis. Note – The figure shows the dynamics of herding behaviour in India, Hong Kong, Indonesia, Malaysia, South Korea, Vietnam, China, and Singapore in pre-crisis, during and post-crisis subsamples where Singapore's volatility has been taken as a proxy for market wide volatility. During the earlier part of the pandemic, herding was not very prominent in any of the countries but with progression, herding became very pronounced during the pandemic and this trend continued in a reduced capacity even after the severity of the pandemic reduced.

Table 1 reports the results of estimating the herding regression for all countries in Eq. (1), there is no presence of any herd formation in our overall data set in China, Singapore, Hong Kong, and India. However, there is significant herding present in Vietnam, Malaysia, South Korea, and Indonesia in pre-crisis period. Figure 1 during our subsample analysis, we find that investors were motivated to herd more strongly during pandemic time in Vietnam, India, and Indonesia while herding was prevalent before covid in only South Korea, Indonesia, and Malaysia. Vietnam was the only country to report post-crisis herding suggesting prolonged effects of the crisis on the investor behaviour. These findings seem to be in contrast with results obtained in the whole sample providing evidence that herding is a short-lived phenomenon.

**Table 1:** Herding Estimates of the whole sample, pre-crisis, during crisis and post crisis.

	**Whole Period**	**Pre-Crisis**	**During Crisis**	**Post -Crisis**
**China**				
α	0.019** (0.100)	0.012* (34.800)	0.013* (31.220)	0.320** (2.500)
$\gamma_{1}$	0.025 (-0.270)	-0.015 (-0.930)	0.021*** (1.040)	0.039*** (1.380)
$\gamma_{2}$	-0.099 (-0.270)	0.126 (0.013)	0.159* (2.860)	-0.895 (-0.690)
$\gamma_{3}$	1.110** (2.550)	4.355** (3.380)	0.687 (0.620)	1.918** (1.380)
Adj R^2^	0.081	0.265	0.164	0.077**
F-Stat	33.620	58.940	16.800	11.470
**Vietnam**				
α	0.016** (22.170)	0.015** (10.080)	0.173** (30.740)	0.020** (21.890)
$\gamma_{1}$	0.025** (0.970)	0.045** (0.690)	0.028*** (1.40)	-0.003** (-0.130)
$\gamma_{2}$	0.496* (5.950)	0.376*** (1.940)	0.378* (5.620)	0.424* (4.810)
$\gamma_{3}$	-0.859 (-0.550)	3.194** (0.780)	-0.281 (-0.220)	-1.137 (-0.750)
Adj R^2^	0.139	0.062	0.446	0.238
F-Stat	62.600	11.950	67.970	41.500
**South Korea**				
α	0.011* (43.400)	0.010* (30.780)	0.012* (23.900)	0.011* (18.750)
$\gamma_{1}$	0.054* (4.410)	0.030*** (1.340)	0.047* (3.010)	0.053** (1.990)
$\gamma_{2}$	0.327* (10.030)	0.325* (4.790)	0.183* (3.600)	0.316* (2.830)
$\gamma_{3}$	0.064 (1.010)	-3.525*** (-1.340)	2.395* (3.100)	5.314*** (1.340)
Adj R^2^	0.278	0.100	0.499	0.226
F-Stat	145.810	19.060	82.830	38.650
**Singapore**				
α	0.007* (22.530)	0.007* (22.240)	0.010* (7.970)	0.006* (17.000)
$\gamma_{1}$	0.007*** (1.190)	0.014** (2.030)	0.009 (0.640)	-0.010** (-1.110)
$\gamma_{2}$	0.817* (53.160)	0.793* (47.990)	0.812* (17.270)	0.816* (26.810)
$\gamma_{3}$	0.894* (9.330)	0.958* (8.190)	0.861* (3.390)	1.078* (2.820)
Adj R^2^	0.945	0.962	0.933	0.947
F-Stat	6630.080	4270.0	1167.910	2120.310
**Malaysia**				
α	0.0138* (8.720)	0.008*** (1.690)	0.016* (18.250)	-0.016* (25.240)
$\gamma_{1}$	0.254*** (1.830)	1.058* (2.210)	0.059*** (1.460)	-0.016 (-0.390)
$\gamma_{2}$	0.738* (3.680)	2.670** (1.860)	0.476* (3.900)	0.363** (1.990)
$\gamma_{3}$	0.736* (3.380)	-51.690 (-0.640)	2.162 (0.840)	4.753 (0.430)
Adj R^2^	0.748	0.018	0.285	0.102
F-Stat	1132.940	3.960	33.740	15.450
**Indonesia**				
α	0.013* (43.95)	0.013* (20.730)	0.013* (20.720)	0.014* (30.160)
$\gamma_{1}$	0.080* (4.780)	0.014 (0.360)	0.117* (4.880)	0.079* (2.860)
$\gamma_{2}$	0.378* (9.200)	0.360** (2.570)	0.516* (7.820)	0.250* (2.710)
$\gamma_{3}$	-0.401 (-0.520)	-4.722 (-0.810)	-2.318** (-2.320)	2.958 (0.870)
Adj R^2^	0.187*	0.036	0.412*	0.145
F-Stat	86.110	7.040	57.030	21.490
**Hong Kong**				
α	0.015* (7.900)	0.012* (40.590)	0.014* (24.870)	0.020* (3.700)
$\gamma_{1}$	0.048 (0.520)	0.051* (3.530)	0.068* (3.350)	0.055 (0.230)
$\gamma_{2}$	0.159*** (1.120)	0.118** (2.290)	-0.025 (-0.320)	0.024 (0.0700)
$\gamma_{3}$	0.855*** (1.120)	3.632** (2.160)	6.496* (3.480)	0.991** (2.240)
Adj R^2^	0.311	0.179	0.235	0.312
F-Stat	169.970	36.740	26.210	57.670
**India**				
α	0.010* (56.100)	0.011* (36.520)	0.011* (23.710)	0.011* (35.610)
$\gamma_{1}$	0.043* (4.500)	0.014 (0.720)	0.058* (3.540)	-0.005 (-0.370)
$\gamma_{2}$	0.268* (12.180)	0.076*** (1.370)	0.343* (7.940)	0.056*** (1.120)
$\gamma_{3}$	0.412*** (1.360)	8.663* (4.720)	-0.467 (-0.950)	3.919** (2.560)
Adj R^2^	0.335	0.216	0.481	0.143
F-Stat	191.400	45.910	78.400	22.730

*Note*: This table reports the regression results of the Eq. (2)$\text{CSA}D_{t\;}\;=\;\alpha \;+\;\gamma_{1}R_{m,t}\;+\;\gamma_{2}\left|R_{m,t}\right|\;+\;\gamma_{3}{\left(R_{m,t}\right)}^{2}\;+\;\varepsilon_{t}$ in which CSAD_t_ is the cross-sectional standard absolute deviations of returns at time t, α is the intercept, γ_1_ is the linear co-efficient between $R_{m,t}$ and CSAD, γ_2_ is the non-linear coefficient between $R_{m,t}$ and CSAD. $R_{m_{t}}$ is the market return at time t and $\varepsilon_{t}$ is the error term, $\gamma_{3}\;$relates CSAD to ${\left(R_{m,t}\right)}^{2}$. The asterisks *, ** and *** denote statistical significance at 1%, 5% and 10% levels. The values in parenthesis () denote t-statistics.

We also examine asymmetries in extreme market returns in Eq. (3). Table 2 Vietnam and Indonesia report high asymmetries with herd formation being stronger in bullish markets. Negative coefficient $\gamma_{2}^{UP}$ in India, Indonesia, South Korea, Singapore, and Vietnam indicates that investors have a healthy risk appetite during bullish markets. Negative $\gamma_{2}^{DOWN}$coefficients in Indonesia and Vietnam suggest strong herd formation during bearish markets. The deviation between dispersions between $\gamma_{2}^{UP}$ and $\gamma_{2}^{DOWN}$ suggest the urge to resort to “flight to safety” strategy. In China, Malaysia, and Hong Kong $\gamma_{2}^{DOWN}$ values are significantly greater than $\gamma_{2}^{UP}$ implying the reliance of investors on available information.

**Table 2:** Herding estimates during extreme market returns.

Panel A: Lower Market									
Criteria	1%					5%			
Country	α	$Y_{1}^{DOWN}$	$Y_{2}^{DOWN}$	Adj R^2^		α	$Y_{1}^{DOWN}$	$Y_{2}^{DOWN}$	Adj R^2^
China	0.006* (25.417)	0.118* (4.279)	0.876* (31.393)	0.104		0.008* (36.640)	0.124* (5.246)	0.869* (36.521)	0.118
Hong Kong	0.006* (19.184)	0.187* (6.784)	0.787* (28.768)	0.177		0.007* (27.796)	0.196* (9.102)	0.777* (36.432)	0.168
India	0.005* (15.957)	0.130** (2.526)	0.568 (0.843)	0.050		0.010* (26.422)	0.152* (4.049)	0.087 (0.146)	0.059
Indonesia	0.008* (24.984)	0.218* (3.72)	-0.212 (-0.165)	0.090		0.009* (26.995)	0.211* (3.124)	-0.420 (-0.263)	0.082
Malaysia	0.006* (19.184)	0.187* (6.784)	0.787* (28.768)	0.177		0.007* (27.796)	0.196* (9.102)	0.777* (36.432)	0.169
Singapore	0.002* (5.466)	0.3717* (6.937)	2.448* (11.050)	0.370		0.003* (5.489)	0.531* (9.318)	1.784* (8.265)	0.479
South Korea	0.005* (12.512)	0.156*** (1.204)	0.300 (0.057)	0.082		0.007* (19.585)	0.195** (2.278)	0.002 (0.001)	0.093
Vietnam	0.008* (18.084)	0.383* (5.381)	-1.156*** (-1.279)	0.200		0.009* (29.108)	0.511* (13.901)	-2.464* (-4.951)	0.230
Panel B: Upper Market									
Criteria	1%					5%			
Country	α	$Y_{1}^{UP}$	$Y_{2}^{UP}$	Adj R^2^		α	$Y_{1}^{UP}$	$Y_{2}^{UP}$	Adj R^2^

China	0.028* (20.173)	0.293* (6.395)	0.676* (14.834)	0.222		0.021* (32.337)	0.239* (3.136)	0.738* (9.619)	0.205
Hong Kong	0.023* (38.167)	0.584* (32.226)	0.373* (20.743)	0.393		0.019 (0.353)	0.34* (9.026)	0.621* (16.583)	0.352
India	0.020* (26.847)	0.638* (9.502)	-3.506* (-7.166)	0.373		0.017* (24.576)	0.355** (2.024)	0.866 (0.232)	0.299
Indonesia	0.027* (12.468)	1.005* (4.524)	-7.372* (-3.529)	0.165		0.019* (10.953)	0.619* (2.824)	1.183 (0.219)	0.1863
Malaysia	0.228* (38.167)	0.584* (32.226)	0.373* (20.743)	0.393		0.0194* (41.1962)	0.34* (9.026)	0.621 (0.037)	0.352
Singapore	0.020* (12.905)	1.535* (11.402)	-1.563* (-2.929)	0.803		0.013* (22.911)	0.983* (12.837)	0.658** (2.270)	0.828
South Korea	0.021* (18.464)	1.670* (6.1627)	17.142* (-5.446)	0.275		0.0179* (25.403)	0.474* (3.853)	2.650*** (1.125)	0.217
Vietnam	0.034* (10.306)	1.496** (2.420)	-9.317 (-1.080)	0.094		0.026* (18.585)	0.536* (3.732)	-0.814 (-0.315)	0.169

*Note*: This table reports the regression results of the Eqs. (2) and (3) respectively.

${\text{CSAD}}_{t}^{\text{UP}}=\;\alpha +\gamma_{1}^{\text{UP}}\left|R_{m,t}^{\text{UP}}\right|+\gamma_{2}^{\text{UP}}{\left(R_{m,t}^{\text{UP}}\right)}^{2}+\varepsilon_{t}$.

${\text{CSAD}}_{t}^{\text{DOWN}}=\;\alpha +\gamma_{1}^{\text{DOWN}}\left|R_{m,t}^{\text{DOWN}}\right|+\gamma_{2}^{\text{DOWN}}{\left(R_{m,t}^{\text{DOWN}}\right)}^{2}+\varepsilon_{t}$.

Up and down indicate rising ($R_{m,t}$ > 0) and falling ($R_{m,t}\;$< 0) market conditions respectively. $R_{m,t}$is the market return at time t. CSAD is the cross-sectional absolute deviation and is computed as in Eq. (1)- $\text{CSA}D_{t\;}\;=\;\alpha \;+\gamma_{1}R_{m,t}+\;\gamma_{2}\left|R_{m,t}\right|\;+\;\gamma_{3}{\left(R_{m,t}\right)}^{2}\;+\;\varepsilon_{t}$, *, **, and *** denotes significance at 1%, 5% and 10% level. The values in parenthesis () denote t-statistics.

We also test for “anti-herding” sentiment and find that a strong presence can be detected in China, Singapore, and Hong Kong in normal and extreme market conditions due to their highly developed and stable financial market system preventing any strong impact of negative news leading to herding.

## Conclusion

We find conclusive evidence that herding is strongly present in emerging markets compared to developed markets in Asia. We also investigated asymmetries and found that investors tend to herd more during bullish market periods implying that the investors may tend to become “greedy” while harbouring a healthy propensity to take risks. Still, in times of economic distress, they could resort to a “flight to safety” strategy. The study finds that herding seems to be strong during and after Covid-19, which is in contrast with evidence of no strong herding being observed in the overall dataset, lending support to the earlier assertion made by researchers that herding is short-lived. These results suggest increased investor confidence and risk-taking appetite in the markets. Thus, in each Asian market, the herding behaviour has different implications and helps in policy decisions. In the Chinese market, investors behave rationally due to high information disclosure and the non-existence of informational cascades. Whereas, in Vietnam, the investors imitated the actions and thought processes of giant brokers or foreign institutional investors. In South Korea, the high degree of financial stability and the market operates relatively independently of government intervention leading to the absence of herding in South Korea. Herd formation can be said to be partially present in the Malaysian stock market during the pre-crisis period implying that the investors expected government intervention during the crisis. Notably, high sophistication of the financial system and complete transparency in disclosure elements, Singapore does exhibit a decisive “anti-herding” sentiment. However, in Indonesian financial markets, investors seem to be completely reliant on external sources of information due to the weak and opaque information disclosures by the required authorities coupled with the heavy government intervention in the stock markets. Similarly, in the case of Hong Kong, the investors are well informed and hence decisions are rational. Finally, the Indian financial market is faced with high informational cascades and asymmetries; thus, herd formation takes place during the crisis. Our results lend support to the earlier studies studying investor behaviour in Asia and are also helpful for understanding the creation of market bubbles due to herd formation while providing policy guidance for informational cascades and market transparency.

## Declaration of Competing Interest

The authors declare that they have no known competing financial interests or personal relationships that could have appeared to influence the work reported in this paper.

## Data Availability

Data will be made available on request.
